# BCAAs and related metabolic enzymes: partners in crime driving tumor development

**DOI:** 10.3389/fcell.2026.1748587

**Published:** 2026-02-13

**Authors:** Binfan He, Lingxi Li, Ye Liu, Mengmeng Hao, Ling Zhang, Rongzhang He

**Affiliations:** 1 School of Basic Medicine, Institute for Pathogenesis and Clinical Translational Research of Esophageal Cancer, Shanxi Medical University, Taiyuan, Shanxi, China; 2 Translational Medicine Institute, The First People’s Hospital of Chenzhou, Hengyang Medical School, University of South China, Chenzhou, China; 3 Department of Basic Medical Sciences, Qinghai University Medical College, Xining, Qinghai, China

**Keywords:** BCAAs, dietary therapy, immunity, metabolism, targeted inhibitors

## Abstract

Metabolic reprogramming of Branched-chain amino acids (BCAAs)-leucine, isoleucine, and valine-has emerged as a constitutive feature of cancer, extending far beyond their canonical roles in protein synthesis and energy provision. In malignancy, these essential amino acids function as pivotal signaling mediators and epigenetic modulators, thereby propelling tumor progression, facilitating immune evasion, and conferring resistance to therapeutic agents. This review delineates how cancer cells subvert branched-chain amino acid metabolism to fuel anabolic processes, activate oncogenic signaling cascades including mTOR and PI3K/AKT, and remodel the tumor microenvironment. A framework is presented to categorize the differential reliance of various cancers on key catabolic enzymes-BCAT1, BCAT2 and BCKDK-underscoring their therapeutic vulnerability. The paradoxical role of BCAAs in modulating anti-tumor immunity is examined alongside the potential of dietary modulation and the development of pharmacological inhibitors targeting this pathway. Concluding perspectives highlight the trajectory for translating these insights into precision oncology, advocating for biomarker-guided and context-specific therapeutic strategies.

## 1 Introduction

Among numerous diseases, cancer stands as one of the most critical challenges to human health, affecting millions of people worldwide each year and leading to countless fatalities (Siegel et al., 2023). According to the latest IARC/WHO estimates, approximately 20 million new cancer cases and 9.7 million cancer-related deaths occurred globally in 2025, highlighting the immense and growing burden of cancer worldwide (Bray et al., 2024; Liu and Zheng, 2024; Zhang et al., 2025a). Tumor initiation and progression are multifactorial processes involving genetic mutations, environmental exposures, immune evasion and metabolic dysregulation (Yang et al., 2019; Zhou et al., 2017). Metabolism constitutes a foundational pillar of cellular life. Core biological processes-including growth, differentiation, death, and stress adaptation-are intricately governed by metabolites and metabolic enzymes (Pavlova and Thompson, 2016; Xu et al., 2021; O'Sullivan et al., 2019). Emerging evidence further positions metabolites as *bona fide* signaling molecules, capable of modulating cellular signal transduction and participating in diverse processes such as intercellular communication and epigenetic regulation (Bergers and Fendt, 2021; Bayoumi et al., 2020; Li and Wang, 2020).

The aberrant metabolic phenotype of tumors was first described in the 1920s by the German physiologist and physician Otto Warburg, who identified a fundamental difference in glucose utilization between tumor and normal cells (Warburg, 1956). In contrast to normal physiology, tumor metabolism represents a highly complex and dynamically rewired state, characterized by imbalances in multiple metabolites and a comprehensive restructuring of metabolic networks (Xia et al., 2021). Critically, the metabolic activities of both tumor cells and infiltrating immune cells within the tumor microenvironment (TME) play decisive roles in cancer progression and anti-tumor immunity (Chen and Li, 2016). Metabolic reprogramming is established as a hallmark of cancer, conferring upon tumor cells the plasticity to adjust their metabolic flux in response to diverse microenvironmental cues and stresses, thereby promoting survival and proliferation (Xia et al., 2021; Martínez-Reyes and Chandel, 2021). This reprogramming frequently originates from the activation of oncogenes or the inactivation of tumor suppressors, genetic alterations that ultimately drive metabolic remodeling and tumor evolution by dysregulating the expression and activity of key metabolic enzymes (Guo et al., 2025). To fuel their relentless expansion, tumor cells exhibit a pronounced dependency on macromolecular substrates-including glucose, amino acids, and fatty acids-which are indispensable for energy generation and biomass synthesis (Altman et al., 2016; Cheng et al., 2018; An et al., 2022).

In recent years, research in metabolomics and tumor biology has revealed the pivotal roles of amino acid metabolism in cancer development, progression, and treatment response (Vettore et al., 2020; Safrhansova et al., 2022). Amino acids not only serve as building blocks for proteins but also function as critical precursors for energy metabolism and biosynthetic pathways. Dysregulated amino acid metabolism is intimately linked to tumor cell proliferation, invasion, metastasis, and immune escape (Shi et al., 2024). Reprogramming of amino acid metabolism in tumors fuels tumor growth by providing both energy and biosynthetic precursors, while also functioning as signaling molecules to regulate tumor development. This metabolic rewiring plays pivotal roles in maintaining cellular redox homeostasis, driving nucleotide synthesis, and supporting bioenergetics. Notably, restricting the metabolism of specific amino acids presents a promising strategy to therapeutically target the progression of particular tumor types. Among amino acids, Branched-chain amino acids (BCAAs)-leucine, isoleucine, and valine-are essential and cannot be synthesized *de novo* in humans. Beyond serving as fundamental substrates for protein synthesis and muscle maintenance-by supporting anabolic signaling and attenuating proteolysis to promote nitrogen balance and metabolic homeostasis (Kawaguchi et al., 2011; Bifari and Nisoli, 2017; Bo and Fujii, 2024). Branched-chain amino acids (BCAAs) are also actively oxidized in peripheral tissues such as skeletal muscle, adipose tissue, and the liver. This catabolic pathway channels intermediates into the tricarboxylic acid (TCA) cycle, thereby contributing to cellular ATP production (Choi et al., 2024; Mann et al., 2021). Importantly, BCAAs metabolism extends beyond mere fuel supply; it is intimately linked to nutrient-sensitive signaling networks. Leucine, in particular, functions as a potent activator of the mechanistic target of rapamycin complex 1 (mTORC1), a central regulator that integrates nutrient availability with cellular growth and autophagic flux (Choi et al., 2024; Di Malta and Ballabio, 2018). Furthermore, the transamination of BCAAs yields metabolites such as glutamate and branched-chain ketoacids, which participate in glial-neuronal metabolic communication and thereby influence neurotransmitter synthesis (Dimou et al., 2022; Kadowaki and Knox, 1982).

Thus, through their dual roles in both bioenergetics and signal transduction, BCAAs emerge as critical players in maintaining systemic metabolic and functional equilibrium. Intriguingly, BCAAs have been shown to inhibit tumor cell migration and metastasis by downregulating N-cadherin expression (Chi et al., 2022). Nonetheless, the role of BCAAs in cancer remains controversial, as some studies demonstrate that their catabolism can promote tumor growth and progression in a dose-dependent manner (Li et al., 2020), while other studies demonstrate that BCAAs can suppress tumor development by enhancing anti-tumor immune responses, as observed in models of pancreatic cancer and triple-negative breast cancer (Chi et al., 2022; Lei et al., 2020; Yao et al., 2023). Consequently, targeting enzymes involved in BCAAs catabolism or adopting BCAAs-enriched dietary regimens has emerged as a potential therapeutic avenue (Lei et al., 2020). However, given the dual-faced role of BCAAs in cancer, translating these strategies into clinical practice may encounter substantial challenges and limitation (Dimou et al., 2022).

This review synthesizes the role of branched-chain amino acid (BCAAs) metabolism in cancer. We detail how BCAAs metabolic reprogramming promotes tumor progression and immune evasion, examine its diagnostic potential, and evaluate therapeutic strategies-including enzyme inhibition and dietary modulation-that target this pathway to suppress tumor growth and enhance therapy response. We also discuss the clinical prospects and challenges of these approaches.

## 2 BCAAs metabolism in physiology

**FIGURE 1:**
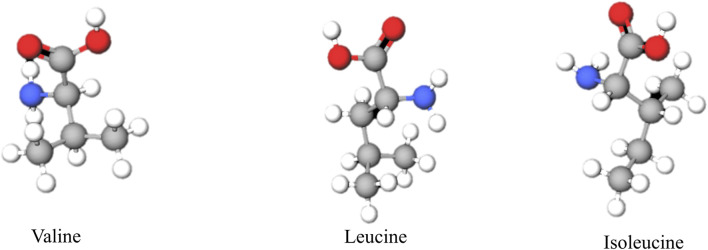
Chemical structures of Branched-chain amino acids. Ball - and - stick models illustrating the chemical structures of branched - chain amino acids: valine, leucine, and isoleucine.

**FIGURE 2:**
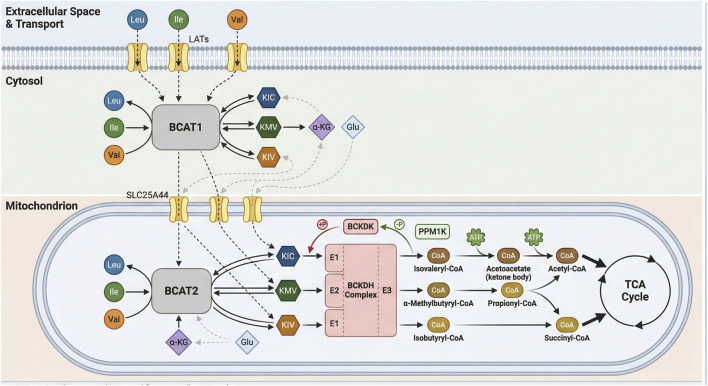
Schematic representation of branched-chain amino acid (BCAAs) metabolism and regulation. This schematic illustrates the compartmentalized BCAA catabolic pathway across the cytosol and mitochondria. In the extracellular space, the L-type amino acid transporter (LATs) imports leucine (Leu), isoleucine (Ile), and valine (Val) into the cytosol. In the cytosol, BCAT1 catalyzes the transamination of BCAAs to generate their corresponding α-keto acids (α-KIC, α-KMV, α-KIV) and glutamate (Glu). These α-keto acids are then transported into the mitochondrial matrix via the SLC25A44 transporter. Within the mitochondria, BCAT2 completes the transamination of BCAAs, producing α-keto acids that are further oxidized by the branched-chain α-keto acid dehydrogenase (BCKDH) complex. The BCKDH complex, regulated by phosphorylation (via PPM1K) and feedback inhibition, generates acyl-CoA intermediates: isovaleryl-CoA, 2-methylbutyryl-CoA, and isobutyryl-CoA. These intermediates are channeled into downstream metabolic pathways, including the production of ketone bodies and acetyl-CoA/propionyl-CoA, which feed into the tricarboxylic acid (TCA) cycle to support cellular energy metabolism and biosynthesis.

Branched-chain amino acids (BCAAs)-leucine, isoleucine, and valine-are essential nutrients that support protein synthesis, energy homeostasis, and metabolic signaling (Figure 1) (Bo and Fujii, 2024; Choi et al., 2024). Leucine is abundantly present in proteins, and its breakdown-whether from exogenous dietary sources or endogenous sources such as muscles-releases a substantial amount of this amino acid, such as during metabolic turnover (Kamphorst et al., 2015). Their transport across both the intestinal epithelium and the plasma membrane is facilitated by specific transporters, namely, members of the L-type amino acid transporter (LATs) (Verkerke et al., 2024). Intracellular BCAAs are catabolized via two compartmentalized pathways: one involves mitochondrial import via Solute Carrier Family 25 Member 44 (SLC25A44) followed by transamination and degradation, and the other involves cytosolic transamination. These initial transamination steps are catalyzed by the respective branched-chain amino acid transaminase (BCAT) isoforms-BCAT1 in the cytosol and BCAT2 in mitochondria (Kadowaki and Knox, 1982; Ichihara, 1975). BCAT1 is predominantly expressed in neuronal tissues and immune cells, such as activated T lymphocytes and macrophages. In contrast, BCAT2 has a broader tissue distribution and is expressed in most tissues, with the highest levels found in skeletal muscle, followed by the kidney, and the lowest in the liver (Bledsoe et al., 1997; Suryawan et al., 1998). The BCAT1 and BCAT2 transfer the amino group from BCAAs to α-ketoglutarate (α-KG), yielding glutamate and branched-chain α-keto acids (BCKAs). Leucine, valine, and isoleucine are catalyzed to form α-ketoisocaproate (KIC), α-ketoisovalerate (KIV), and α-keto-β-methylvalerate (KMV), respectively. Notably, to replenish α-ketoglutarate and Branched-chain amino acids (BCAAs), the transaminases BCAT1 and BCAT2 transfer the nitrogen from glutamate back to a branched-chain α-keto acid (Dimou et al., 2022). This latter reaction is catalyzed by the branched-chain α-ketoacid dehydrogenase (BCKDH) complex, which comprises three functional components: the thiamine-dependent decarboxylase (E1), consisting of α- and β-subunits encoded by the BCKDHA and BCKDHB, respectively; the dihydrolipoyl transacylase (E2), encoded by the DBT; and the dihydrolipoamide dehydrogenase (E3), encoded by the DLD (Neinast et al., 2019; Li Z. et al., 2024). BCKDH has been reported as the rate-limiting enzyme in BCAAs catabolism. Its overall activity is dynamically regulated by BCKDK-mediated phosphorylation and Protein Phosphatase, Mg^2+^/Mn^2+^ Dependent 1K (PPM1K)-mediated dephosphorylation through reversible phosphorylation (Paxton and Harris, 1982; Pettit et al., 1978; Patel et al., 2014). BCKDH yielding corresponding branched-chain acyl-CoA derivatives. Specifically, leucine catabolism generates acetyl-CoA and acetoacetate, valine yields propionyl-CoA, and isoleucine produces both acetyl-CoA and propionyl-CoA. Propionyl-CoA is subsequently converted to succinyl-CoA (Mann et al., 2021; Du et al., 2022). These intermediates are ultimately converted into acetyl-CoA or succinyl-CoA, which enter the tricarboxylic acid (TCA) cycle to support adenosine triphosphate (ATP) production (Figure 2) (Du et al., 2022).

## 3 BCAAs metabolism in cancer

### 3.1 Reprogramming of BCAAs metabolism from catabolism to anabolic signaling

In cancer, BCAAs metabolism is rewired to drive anabolic growth. Tumors frequently upregulate BCAT1, enhancing the conversion of BCAAs to BCKAs in the cytosol (Qian et al., 2023). Concurrently, BCKDH activity is often suppressed through dysregulation of its regulatory enzymes-typically via enhanced BCKDK activity or loss of PPM1K function-leading to marked accumulation of BCKAs (Lu et al., 2024; Du et al., 2018). These metabolites function as biosynthetic precursors for protein and lipid synthesis, directly facilitating biomass accumulation in proliferating cells. This metabolic shift is frequently accompanied by aberrant overexpression of transporters such as *LAT1* and *SLC25A44*, thereby supplying ample substrate to fuel BCAT-driven metabolic flux (Schroeder et al., 2024; Yoneshiro et al., 2019).

Notably, accumulated BCKAs contribute to sustained activation of the mechanistic target of rapamycin complex 1 (mTORC1), further stimulating cell growth and establishing a feed-forward loop that reinforces metabolic reprogramming and malignant progression (Di Malta and Ballabio, 2018). Thus, tumor cells divert BCAAs catabolism from energy generation toward biosynthesis and oncogenic signaling, unveiling this pathway as a key metabolic vulnerability in cancer.

### 3.2 Classification of tumor dependency on BCAAs metabolism

The reliance of various cancers on BCAAs metabolism can be stratified into three distinct archetypes, providing a clarifying framework that moves beyond a simple listing.

#### 3.2.1 Tumors with strong (obligate) dependency

Cancers such as glioblastoma (GBM) and acute myeloid leukemia (AML) exhibit a non-redundant, cell-autonomous reliance on BCAAs metabolic reprogramming, primarily driven by *BCAT1* overexpression (Hattori et al., 2017). In these malignancies, BCAT1 activity is a critical engine for sustaining proliferation, stemness, and survival (Hattori et al., 2017; Zhang et al., 2022). Inhibition of this pathway presents a clear and direct therapeutic vulnerability, as evidenced by synthetic lethality upon BCAT1 knockdown or pharmacological inhibition.

#### 3.2.2 Tumors with tissue-of-origin dependency

**FIGURE 3:**
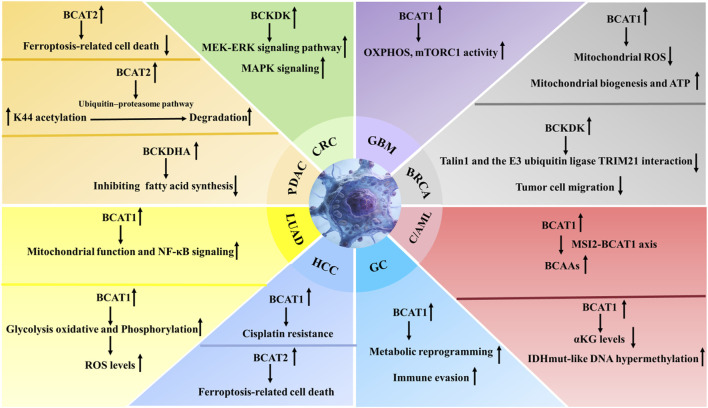
BCAAs metabolism-related enzyme (BCAT1/2, BCKDK, BCKDHA) regulatory networks and functional outcomes across human cancers. This schematic integrates tissue-specific (color-coded by cancer type: e.g., CRC, PDAC, GBM, BRCA, GC, HCC, LUAD, CAML) regulatory circuits of branched-chain amino acid (BCAAs) metabolism enzymes and their downstream effects in tumorigenesis. Arrows (↑/↓) denote upregulation/downregulation of the enzyme; dashed lines link enzymatic activity to functional consequences (e.g., signaling, metabolism, cell death, therapy resistance).

This category highlights how the tissue microenvironment dictates specific BCAAs utilization strategies. For instance, lung adenocarcinomas actively scavenge extracellular BCAAs as a nitrogen source to support anabolic needs (Mayers et al., 2016). In contrast, pancreatic ductal adenocarcinomas (PDAC) exhibit a more complex, parasitic relationship with their stroma, relying on BCAT2-mediated metabolism and potentially on BCAAs-derived metabolites from the tumor microenvironment (Li et al., 2020; Lei et al., 2020). This fundamental difference underscores that therapeutic targeting must consider the ecological context of the tumor (Figure 3).

#### 3.2.3 Tumors with contextual duality or opposing roles

In cancers such as breast cancer (particularly triple-negative) and colorectal cancer (CRC), BCAAs metabolism plays a complex, double-edged role. Within tumor cells, it fuels anabolic growth and proliferation, with BCAT1 being essential in TNBC models (Huang et al., 2025; Thewes et al., 2017). Systemically, however, it simultaneously modulates anti-tumor immunity-potentially enhancing CD8^+^ T cell function (Chi et al., 2022; Yao et al., 2023), while also supporting immunosuppressive regulatory T cells (Tregs) (Ikeda et al., 2017). This paradox creates a therapeutic dilemma where inhibiting tumor-intrinsic BCAAs catabolism may have unintended consequences on the immune response (Dimou et al., 2022), necessitating highly context-aware strategies.

This stratification clarifies that BCAAs metabolism is not a uniformly similar pathway across cancers but a context-dependent vulnerability. It enhances the review’s guidance by emphasizing that future therapeutic strategies-whether involving enzyme inhibitors, dietary modulation, or their combination with immunotherapy-must be precisely tailored according to these dependency archetypes, integrating tumor lineage, genetic drivers, and the specific immune and metabolic landscape of the tumor microenvironment.

### 3.3 BCAAs metabolism as an integrative node in tumor progression: a coordinating framework

**FIGURE 4:**
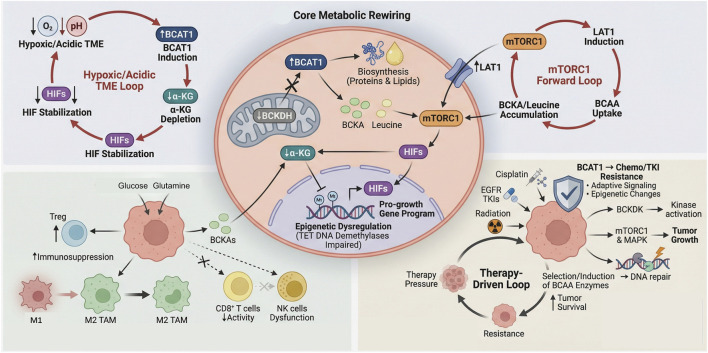
Core metabolic rewiring in the tumor microenvironment drives immunosuppression, therapy resistance, and tumor survival. This schematic outlines the multifaceted roles of BCAT1 in tumor pathophysiology. In the core panel, BCAT1 catabolizes branched-chain amino acids (BCAAs) to fuel biosynthesis, elevate intracellular leucine, and activate mTORC1-LAT1 signaling, creating a feed-forward BCAA uptake cycle. Concurrently, BCAT1-mediated α-ketoglutarate (α-KG) depletion stabilizes HIF-1α in hypoxic/acidic tumor microenvironments (TMEs), driving a pro-growth transcriptional program. In the immunosuppressive TME (bottom left), BCAT1-dependent metabolic competition restricts nutrient availability for effector T cells, polarizes macrophages to an M2 phenotype, expands regulatory T cells, and impairs CD8^+^ T-cell function. In therapy resistance (bottom right), BCAT1 activates adaptive signaling and DNA repair pathways to counteract chemotherapy, targeted therapy, and radiation, with therapy-induced selective pressure further upregulating BCAT1 to reinforce a pro-survival, therapy-driven loop. Hypoxia and acidosis in the TME (top left) initiate this cascade by stabilizing HIF-1α and inducing BCAT1 expression.

Reprogramming of BCAAs metabolism operates as a central signaling hub. This hub dynamically coordinates tumor growth, immune evasion, and therapy resistance through a network of interlocking feedback loops (Figure 4). Initiation of this circuit commonly stems from tumor-intrinsic metabolic rewiring, typified by upregulation of BCAT1 and/or suppression of the BCKDH complex. This diversion of BCAAs from oxidative catabolism toward biosynthetic pathways serves a dual role: it supplies precursors for macromolecule synthesis (e.g., branched-chain α-keto acids for protein and lipid production) and generates metabolite-derived oncogenic signals. A pivotal consequence of elevated BCAT1 activity is the depletion of cytosolic α-ketoglutarate (α-KG). This reduction impairs the function of α-KG-dependent dioxygenases, such as the TET family of DNA demethylases, leading to epigenetic dysregulation (e.g., DNA hypermethylation) and stabilization of hypoxia-inducible factors (HIFs). Consequently, a pro-growth transcriptional program becomes entrenched (Hattori et al., 2017; Meurs and Nagrath, 2022; Raffel et al., 2017). In parallel, accumulating BCKAs and leucine provide sustained activation of mTORC1, establishing a feed-forward loop that further induces the expression of BCAAs transporters like *LAT1*, thereby amplifying substrate uptake and reinforcing the metabolic shift (Di Malta and Ballabio, 2018; Mayers et al., 2016).

The altered metabolic state of the cancer cell extends its influence to remodel the tumor microenvironment (TME). Nutrient competition and the release of specific metabolites, including BCKAs, enact context-dependent immunomodulatory effects. These changes can potentiate the immunosuppressive activity of regulatory T cells (Tregs) and drive the polarization of tumor-associated macrophages toward a pro-tumorigenic M2 phenotype. Simultaneously, they contribute to a metabolically restrictive niche that compromises the effector functions of CD8^+^ T cells and natural killer (NK) cells (Ikeda et al., 2017; Zhang et al., 2024; Zhang et al., 2023). Collectively, these actions reshape the TME into an immunosuppressive ecosystem conducive to immune escape.

This integrated metabolic network also constitutes a direct engine of therapy resistance. The very enzymes that propel tumor growth and immune suppression simultaneously confer resilience against therapeutic agents. For example, BCAT1 has been shown to mediate resistance to conventional chemotherapeutics like cisplatin and to targeted agents such as EGFR tyrosine kinase inhibitors, achieved through adaptive signaling and epigenetic modifications (Zhang et al., 2024; Sjögren, 2021). Correspondingly, activation of BCKDK promotes tumor progression via mTORC1 and MAPK signaling pathways and has been linked to radioresistance by enhancing DNA damage repair mechanisms. This connection implicates BCAAs metabolism in treatment failure across diverse therapeutic modalities (Bo et al., 2025; Wang Y. et al., 2021; Liu et al., 2025).

These processes are not linear but are interconnected through reinforcing feedback loops. The hypoxic and acidic conditions of the TME, frequently a product of rapid tumor expansion, can themselves induce *BCAT1* expression via HIF signaling (Meurs and Nagrath, 2022). The subsequent BCAT1-mediated depletion of α-KG then further stabilizes HIFs, creating a vicious cycle that perpetuates both metabolic and epigenetic reprogramming. In a similar vein, therapeutic pressure can select for cell populations with upregulated BCAAs pathway enzymes or directly induce their expression, thereby driving acquired resistance.

In summary, BCAAs metabolic reprogramming not as a peripheral supportive feature, but as a core orchestrator of malignant progression. It functions as a self-amplifying network that synergistically augments tumor cell fitness, dismantles anti-tumor immunity, and subverts therapeutic efficacy. Disruption of this network-via targeted enzyme inhibition, dietary modulation, or combination strategies informed by specific tumor dependency archetypes-represents a promising multidimensional therapeutic approach. Such strategies aim to simultaneously intercept multiple hallmark capabilities of cancer. Future research and clinical translation must rigorously account for the interconnected nature of this pathway to design effective and context-specific interventions.

## 4 The role of BCAT1 and BCAT2 in cancer

### 4.1 Integrated role of BCAT1 and BCAT2 in BCAAs metabolism and signaling

**FIGURE 5:**
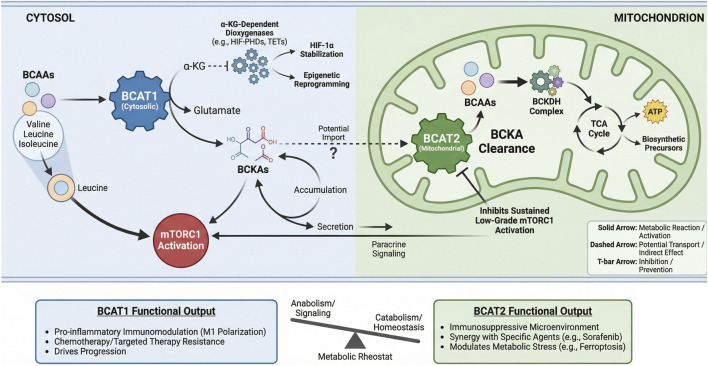
Spatial regulation of BCAAs metabolism by BCAT1 and BCAT2 dictates functional Output. BCAT1 (cytosolic) and BCAT2 (mitochondrial) compartmentalize branched-chain amino acid (BCAAs) metabolism, leading to distinct functional consequences. Cytosolic BCAT1 activity consumes α- KG to produce glutamate, supporting α-KG-dependent dioxygenases and generating BCKAs that can activate mTORC1 or signal in a paracrine manner. Its functional output promotes inflammation, therapy resistance, and disease progression. Mitochondrial BCAT2 initiates BCKA catabolism via the BCKDH complex, feeding the TCA cycle and preventing sustained mTORC1 activation. BCAT2 function fosters an immunosuppressive microenvironment and modulates metabolic stress responses.

BCAT1 and BCAT2 constitute a spatially compartmentalized regulatory circuit that fundamentally dictates BCAAs metabolic fate. Their distinct subcellular localization-cytosolic versus mitochondrial-establishes a metabolic bifurcation point (Figure 5) (Ananieva and Wilkinson, 2018). BCAT1 primarily channels BCAAs toward anabolic and signaling outputs: its transamination reaction depletes α-ketoglutarate (α-KG), influencing epigenetics and hypoxia responses, while elevating leucine to directly activate mTORC1. The resultant branched-chain α-keto acids (BCKAs) may accumulate or be secreted, acting as paracrine signals (Wegermann et al., 2018). In contrast, BCAT2 commits BCKAs to oxidative catabolism in the mitochondria, thereby controlling their clearance rate and preventing aberrant accumulation that can lead to sustained, low-grade mTORC1 activation (Sjögren, 2021). This dynamic interplay between the two enzymes regulates the balance between cytosolic α-KG availability, BCKA pools, and downstream anabolic signaling. Functionally, BCAT1 often drives pro-inflammatory immune modulation and confers resistance to chemotherapy and targeted therapies (Mayers et al., 2016; Zhang et al., 2024; Zheng et al., 2016). Conversely, BCAT2 frequently supports an immunosuppressive tumor microenvironment and its modulation can synergize with specific agents to induce metabolic stress (Cai et al., 2023; Wang K. et al., 2021). Thus, the BCAT1/BCAT2 axis acts as a metabolic rheostat, where the relative activity of each enzyme determines whether BCAAs fuel growth signals or are catabolized for homeostasis-a balance frequently disrupted in cancer to support progression and therapy resistance.

### 4.2 The role of BCAT1 in cancer

Branched-chain amino acid transaminase 1 (BCAT1) is frequently overexpressed across multiple cancer types (Table 1), where it has been functionally linked to enhanced tumor proliferation, therapy resistance, and immune evasion. In gastric cancer, elevated BCAT1 activates PI3K/AKT/mTOR pathway, drives tumor proliferation, invasion, and angiogenesis, correlating with unfavorable clinical outcomes (Shu et al., 2021; Xu et al., 2018). Similarly, in oral carcinomas, BCAT1 promotes cell proliferation and migration through activation of the PI3K/Akt signaling axis (Yuan et al., 2025). Within triple-negative breast cancer models, BCAT1 appears indispensable for sustaining tumor growth; its inhibition induces apoptosis via suppression of the SHOC2–RAS–ERK pathway induce apoptosis, highlighting a potential therapeutic vulnerability (Huang et al., 2025). Consistent with these findings, *BCAT1* expression is markedly higher in breast tumor tissues compared to adjacent normal tissue, where it supports tumor cell proliferation by promoting mitochondrial biogenesis through an mTOR-dependent mechanism (Thewes et al., 2017). Further evidence indicates that in chronic myeloid leukemia (CML), BCAT1 catalyzes the conversion of branched-chain α-keto acids (BCKAs) to BCAAs, leading to mTOR pathway activation independent of AKT signaling and thereby accelerating disease progression (Hattori et al., 2017). Experimental inhibition of BCAT1 with gabapentin significantly curbs proliferation in human colorectal cancer cells (Jedi et al., 2018). In glioblastoma, knockdown of BCAT1 compromises oxidative phosphorylation (OXPHOS), mTORC1 activity, and nucleotide synthesis. Supplementation with α-ketoglutarate synergistically potentiates these impairments, leading to tumor cell death (Zhang et al., 2022; Meurs and Nagrath, 2022). Under hypoxic conditions, hypoxia-inducible factor (HIF) regulates BCAAs metabolic reprogramming via BCAT1, and gabapentin treatment substantially impairs colony-forming capacity in glioma cell lines (Zhang et al., 2021). It should be noted, however, that *BCAT1* is not universally overexpressed; in certain non-small cell lung cancer (NSCLC) subtypes, its expression remains relatively low, and inhibition exerts no significant effect on tumor growth (Mayers et al., 2016).

**TABLE 1:** The role of BCAT1 in cancer.

Regulator	Cancer type	Mechanism	References
BCAT1	GC	Activates PI3K/AKT/mTOR Pathway	Shu et al. (2021)
BCAT1	LUAD	Drives resistance to EGFR-TKIs through epigenetic modulation of glycolytic pathways	Zhang et al. (2024)
BCAT1	OSCC	Activates PI3K-Akt signaling pathway	Yuan et al. (2025)
BCAT1	TNBC	BCAT1 indirectly controlled expression of the cell cycle inhibitor p27Kip1 thereby affecting pRB	Bo and Fujii (2024)
BCAT1	TNBC	Inhibition of BCAT1 suppresses SHOC2 translation and the downstream SHOC2-participating RAS-ERK signaling pathway	Huang et al. (2025)
BCAT1	NSCLC	High levels of BCAT1 depleted α-ketoglutarate (α-KG) and promoted expression of *SOX2*	Lei et al. (2020)
BCAT1	AML	BCAT1 restricts αKG levels in AML stem cells leading to IDHmut-like DNA hypermethylation	Kadowaki and Knox (1982)
BCAT1	CML	Enhances TKI resistance	Jiang et al. (2025)
BCAT1	CML	Catalyzes conversion of BCKAs to BCAAs, leading to mTOR pathway activation and accelerated disease progression	Hattori et al. (2017)
BCAT1	GBM	The loss of BCAT1 impaired oxidative phosphorylation mTORC1 activity, and nucleotide biosynthesis	Zhang et al. (2022)
BCAT1	BRCA	Promote mitochondrial biogenesis through mToR-dependent mechanism	Thewes et al. (2017)
BCAT1	HCC	Confers resistance to cisplatin	Zheng et al. (2016)

Beyond promoting tumor progression, BCAT1 also mediates resistance to chemotherapy. In hepatocellular carcinoma (HCC), it confers resistance to cisplatin (Zheng et al., 2016) while in lung adenocarcinoma, BCAT1 drives resistance to EGFR tyrosine kinase inhibitors (TKI) through epigenetic modulation of glycolytic pathways (Zhang et al., 2024). A parallel role is observed in CML, where BCAT1 enhances TKI resistance (Jiang et al., 2025). Collectively, these observations position BCAT1-driven metabolic reprogramming as a recurrent mechanism underlying acquired therapy resistance in diverse malignancies.

### 4.3 The role of BCAT2 in cancer

BCAT2, another key enzyme in BCAAs metabolism, is essential for the development and progression of various cancers (Table 2). BCAT2 enhances cellular BCAAs uptake, maintains metabolic homeostasis, and supports mitochondrial respiration. Notably, in mouse models of pancreatic cancer, exogenous BCAAs promote ductal organoid growth, whereas supplementation with BCKAs and nucleobases reverses the antitumor effects of BCAT2 inhibition (Li et al., 2020). In PDAC patient specimens and murine models, BCAT2 is consistently upregulated; its targeted knockdown in pancreatic tissue effectively suppresses the formation of pancreatic intraepithelial neoplasia (Lei et al., 2020). Post-translational regulation of BCAT2 involves acetylation at lysine 44 (K44), which promotes degradation via the ubiquitin-proteasome system, thereby attenuating BCAAs catabolism and restraining pancreatic cancer cell growth (Lei et al., 2020). In melanoma, BCAT2 fosters lipogenesis by depleting acetyl-CoA and inhibiting histone acetylation at promoter regions of lipogenic enzymes such as fatty acid synthase and ATP-citrate lyase (Tian et al., 2023a). In colorectal cancer, loss of BCAT2 leads to sustained mTORC1 activation and accelerates tumor progression (Kang et al., 2024). In prostate cancer, BCAT2 interacts with poly(C)-binding protein 1 through leucine 239, suppressing autophagy-dependent apoptosis and ferroptosis via the PI3K/AKT pathway (Mei et al., 2025). Furthermore, BCAT2 contributes to an immunosuppressive tumor microenvironment by downregulating pro-inflammatory chemokines and limiting cytotoxic lymphocyte infiltration, which in turn drives resistance to anti-PD-1/PD-L1 therapies (Cai et al., 2023). In HCC, BCAT2 modulates ferroptosis and exhibits synergistic antitumor activity when combined with sorafenib and sulfasalazine (Wang K. et al., 2021).

**TABLE 2:** The role of BCAT2 in cancer.

Regulator	Cancer type	Mechanism	References
BCAT2	PAAC/PDAC	K44 acetylation promotes degradation via the ubiquitin-proteasome system, attenuating BCAA catabolism and restraining tumor growth	Lei et al. (2020)
BCAT2	SKCM	Promotes lipogenesis by depleting acetyl-CoA and inhibiting histone acetylation at promoters of lipogenic enzymes	Tian et al. (2023a)
BCAT2	CRC	Loss of BCAT2 leads to sustained mTORC1 activation and accelerates tumor progression	Kang et al. (2024)
BCAT2	PRAD	Interacts with PCBP1 via leucine 239 to suppress autophagy-dependent apoptosis and ferroptosis through the PI3K/AKT pathway	Mei et al. (2025)
BCAT2	HCC	Modulates ferroptosis and exhibits synergistic antitumor activity when combined with sorafenib and sulfasalazine	Wang K. et al. (2021)

In summary, BCAT1 upregulation is observed in a wide spectrum of cancers-including gastric, oral, nasopharyngeal, breast, gliomas, certain NSCLC subtypes, and AML-where it promotes progression through PI3K/Akt, SHOC2–RAS–ERK, and mTOR signaling pathways. BCAT2, by contrast, has been implicated in NSCLC, PDAC, melanoma, HCC, and prostate cancer, where it accelerates tumorigenesis via mechanisms such as K44 acetylation-mediated degradation, ferroptosis regulation, and activation of mTORC1 and PI3K/AKT signaling. Although both enzymes generally exert pro-tumor effects, their roles can diverge depending on context. In AML, co-upregulation of BCAT1 and BCAT2 triggers BCAAs metabolic reprogramming and fuels disease progression. Interestingly, in colorectal cancer, the two enzymes appear to function antagonistically: BCAT1 inhibition impairs proliferation, whereas BCAT2 loss activates mTORC1 and accelerates disease, suggesting tissue-specific regulatory networks that remain to be fully elucidated. The differential involvement of BCAT1 and BCAT2 in therapy resistance further highlights their clinical relevance. While BCAT1 confers resistance to cisplatin in HCC and to TKIs in lung adenocarcinoma and CML, BCAT2 synergizes with sorafenib and sulfasalazine in HCC. These contrasting profiles imply that tailored therapeutic strategies-combining specific chemotherapy agents with selective inhibition or modulation of BCAT1 or BCAT2-could offer novel avenues for overcoming treatment resistance, particularly in malignancies such as HCC.

## 5 The role of BCKDH–BCKDK–PPM1K complex in cancer

The branched-chain α-ketoacid dehydrogenase complex (BCKDH) is the core enzyme complex for BCAAs catabolism. It is composed of three catalytic components: a heterotetrametric (α2β2) branched-chain α-ketoacid decarboxylase (E1), dihydrolipoyl transacylase (E2), and dihydrolipoyl dehydrogenase (E3). BCKDH directly regulates the oxidative metabolism of BCAAs and energy supply. The BCKDH complex catalyzes the rate-limiting step in BCAAs oxidation and is regulated by phosphorylation via BCKDK (inhibiting) and dephosphorylation via PPM1K (activating) (White et al., 2018; Sivanand and Vander Heiden, 2020; Peng et al., 2020). Reduced *BCKDHA* expression in pancreatic ductal adenocarcinoma (PDAC) suppresses proliferation by inhibiting fatty acid synthesis, suggesting a tumor-suppressive role (Lee et al., 2019). *BCKDHA* expression is markedly upregulated in melanoma tissues and cell lines, where it facilitates tumor progression by enhancing the expression of key lipogenic enzymes, *FASN* and *ACLY* (Tian et al., 2023b). Furthermore, X-ray irradiation induces BCKDHA dephosphorylation, activating branched-chain amino acid (BCAAs) catabolism. The resulting accumulation of BCAAs confers radio resistance by mitigating DNA damage and promoting cancer cell survival (Bo et al., 2025). PPM1K modulates BCAAs levels to regulate CDC20-mediated ubiquitination of MEIS1 and p21, affecting stem cell function in hematological malignancies (Liu et al., 2018). PPM1K is downregulated in pancreatic adenocarcinoma (PAAD) and correlates inversely with EMT and poor prognosis (Zhuang et al., 2023). BCKDH, the core enzymatic complex in BCAAs catabolism regulated by phosphorylation (BCKDK/PPM1K), plays a context-dependent role in cancer. It suppresses tumor growth in pancreatic cancer by inhibiting fatty acid synthesis but promotes progression in melanoma via enhanced lipogenesis. Additionally, its activation confers radio resistance through BCAAs accumulation-mediated DNA damage reduction, and its regulator PPM1K is associated with poor cancer prognosis.

BCKDK is overexpressed in many cancers and promotes growth and proliferation (Maguolo et al., 2022). Its inhibition with BT2 activates BCKDH, lowering BCAAs and BCKA levels (East et al., 2021). In NSCLC, BCKDK activates mTORC1, promoting proliferation and suppressing apoptosis (Wang Y. et al., 2021), potentially through enhancement of Rab1A–mTORC1 signaling (Xue et al., 2023). BCKDK also promotes EMT in CRC via Src-mediated phosphorylation at Y246, increasing its stability and activity (Tian et al., 2020). In HCC, APN mediates BCKDK phosphorylation at S31, activating ERK signaling and promoting metastasis (Zhai et al., 2020). In breast cancer, BCKDK stabilizes Talin1 by interfering with TRIM21-mediated ubiquitination, activating FAK/MAPK signaling and driving metastasis (Xu et al., 2023). Similarly, in ovarian cancer, BCKDK promotes proliferation and migration via MEK/ERK activation (Li H. et al., 2022). Targeting BCKDK may thus offer novel therapeutic opportunities. BCKDK is overexpressed in multiple cancers and promotes tumor growth, proliferation, and metastasis by activating key oncogenic pathways such as mTORC1, ERK, and FAK/MAPK signaling. Its inhibition, for example, with BT2, presents a promising therapeutic strategy by reactivating BCAAs catabolism (Table 3).

**TABLE 3:** The role of the BCKDH–BCKDK–PPM1K complex in cancer.

Regulator	Cancer type	Mechanism	References
BCKDHA	PDAC	Low expression reduces the proliferation rate of pancreatic ductal carcinoma cells by inhibiting fatty acid synthesis	Lee et al. (2019)
BCKDHA	SKCM	BCKDHA contributes to melanoma progression by promoting the expressions of lipogenic enzymes FASN and ACLY	Tian et al. (2023b)
BCKDHA	PAAD	Dephosphorylation-driven BCAAs catabolism activation confers radioresistance in cancer cells	Bo et al. (2025)
PPM1K	AML	PPM1K deficiency led to a notable decrease in MEIS1/p21 signaling to reduce the glycolysis and quiescence of HSCs	Liu et al. (2018)
PPM1K	PAAD	Downregulated and correlates inversely with EMT and poor prognosis	Zhuang et al. (2023)
BCKDK	NSCLC	BCKDK activates mTORC1, promoting proliferation and suppressing apoptosis	Xue et al. (2023)
BCKDK	CRC	BCKDK promotes metastasis of colorectal canceby SrcPhosphorylation at Y246	Tian et al. (2020)
BCKDK	HCC	APN mediates BCKDK phosphorylation at S31,promoting hepatocellular carcinoma metastasis and proliferation via the ERK signaling pathway	Zhai et al. (2020)
BCKDK	BRCA	Stabilizes Talin1 by interfering with TRIM21mediated ubiquitination activating FAK/MAPK signaling and driving metastasis	Xu et al. (2023)
BCKDK	OV	BCKDK promotes proliferation and migration via MEK/ERK activation	Li H. et al. (2022)

## 6 The role of BCAAs in modulating anti-tumor immunity

**FIGURE 6:**
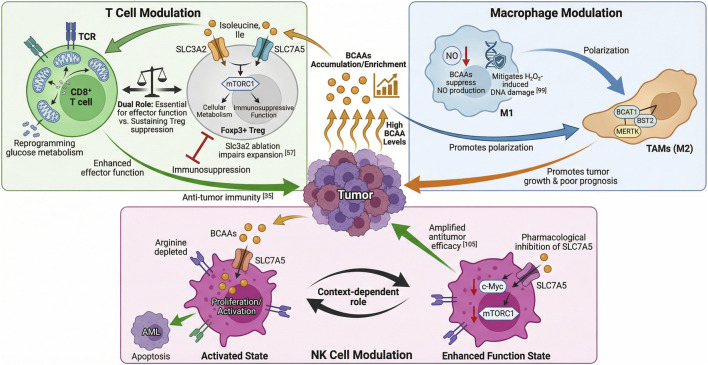
The dual role of branched-chain amino acids (BCAAs) in modulating anti-tumor immunity. This schematic illustrates the differential effects of branched-chain amino acids (BCAAs) on immune cell modulation within the tumor microenvironment. Left Panel: T Cell Modulation – BCAAs promote glucose metabolism reprogramming in CD8^+^ T cells via TCR and mTORC1 signaling, enhancing effector function and antitumor immunity. Middle Panel: Macrophage Modulation – Accumulation of BCAAs in macrophages suppresses nitric oxide (NO) production and mitigates H_2_O_2_-induced DNA damage, but also promotes M2-like polarization, supporting tumor growth and poor prognosis. Right Panel: NK Cell Modulation – BCAAs enhance the functional state of natural killer (NK) cells, contributing to antitumor immunity.

Branched-chain amino acids (BCAAs) and their metabolic reprogramming regulate immune system function by influencing the activity of key immune cells-including T cells, macrophages, and natural killer (NK) cells (Figure 6).

### 6.1 BCAAs metabolism in T cell-mediated tumor immunity

BCAAs play a dual and context-dependent role in tumor progression by modulating T cell function. On one hand, BCAAs-particularly leucine-are essential for immune regulation through metabolic reprogramming and can contribute to antitumor responses, though their precise mechanisms remain incompletely elucidated (Zhou et al., 2025). On the other hand, BCAAs depletion may compromise T cell effector functions, dampening the anti-tumor immune response (Xia et al., 2021). Specific ablation of Slc3a2 in Foxp3^+^ Tregs impairs their *in vivo* expansion by disrupting isoleucine-dependent mTORC1 activation and cellular metabolism; conversely, enhanced BCAA metabolism sustains Treg survival and immunosuppressive function to promote tumor immune evasion (Ikeda et al., 2017). BCAAs accumulation enhances CD8^+^ T cell antitumor immunity by reprogramming glucose metabolism. This mechanism strengthens CD8^+^ T cell effector function, improving responses in lung cancer (Yao et al., 2023). Correspondingly, high BCAAs levels inhibit breast cancer progression and metastasis, likely through boosting CD8^+^ T cell activity (Chi et al., 2022). SLC3A2 and SLC7A5 serve as primary transporters for essential amino acids (O'Sullivan et al., 2019). Within activated T cells, SLC3A2 promotes T cell expansion, whereas SLC7A5 is involved in T cell differentiation, mTORC1 signaling activation, and *c-Myc* expression (Ikeda et al., 2017; Nachef et al., 2021; Kanai, 2022). Notably, inhibition of SLC7A5 compromises the capacity of T cells to eliminate tumor cells. Following T cell receptor activation, human CD4^+^ T cells upregulate both BCAT1 and SLC7A5, thereby enhancing leucine influx and catabolism-a process particularly critical for T helper 17 (Th17) cell responses (Najumudeen et al., 2021).

### 6.2 BCAAs metabolism in macrophage-mediated tumor immunity

Branched-chain amino acids (BCAAs) are implicated in the metabolism and genetics of macrophages. BCAAs significantly suppress nitric oxide production in macrophages, with leucine exhibiting the most pronounced inhibitory effect. Additionally, BCAAs exert a protective role by mitigating hydrogen peroxide-induced DNA damage in macrophages (Lee et al., 2017). BCAAs metabolism also regulates macrophage polarization. In the pancreatic tumor microenvironment, infiltration of M2-type macrophages is markedly elevated which promotes pancreatic tumor growth and is associated with poor disease prognosis (Zhang et al., 2023). BCAT1 collaborates with bone marrow stromal antigen 2 (BST2) and the tyrosine kinase MERTK to facilitate cancer progression by regulating the type 2 polarization of tumor-associated macrophages, offering a potential therapeutic target for pancreatic cancer (Peng et al., 2023). Furthermore, BCAAs accumulation may promote M2-type macrophages, which support tumor growth and immunosuppression, while suppressing M1-type macrophages that possess anti-tumor functions.

### 6.3 BCAAs metabolism in NK cell-mediated tumor immunity

In acute myeloid leukemia (AML), elevated expression of *SLC7A5* enables natural killer (NK) cells to sustain a proliferative and activated state even under arginine-depleted conditions, promoting apoptosis of AML cells (Stavrou et al., 2023). Beyond leukemic contexts, increased levels of Branched-chain amino acids (BCAAs) enhance NK cell function, thereby restraining breast cancer progression and metastasis (Chi et al., 2022). Conversely, in cytokine-activated NK cells, pharmacological inhibition of SLC7A5 lowers c-Myc protein abundance and suppresses mTORC1 signaling, which unexpectedly amplifies their antitumor efficacy (Loftus et al., 2018). Together, these observations highlight the context-dependent role of SLC7A5 and BCAAs metabolism in shaping NK cell activity, offering potential therapeutic avenues for modulating antitumor immunity.

BCAAs metabolism orchestrates a context-dependent immunomodulatory network that differentially regulates the function of major immune cell populations. In T cells, BCAAs exhibit a dual role: while required for maintaining CD8^+^ T cell effector capacity and antitumor immunity, they concurrently reinforce the immunosuppressive activity of regulatory T cells (Tregs), thereby facilitating immune escape. In macrophages, BCAAs-driven metabolic reprogramming promotes polarization toward a pro-tumor M2 phenotype while suppressing the antitumor functions associated with M1 macrophages. With respect to NK cells, although BCAAs availability generally supports cytotoxic function, pharmacological inhibition of the BCAAs transporter SLC7A5 has been observed to unexpectedly enhance antitumor efficacy, revealing a targetable metabolic checkpoint in these cells. Central to this regulatory network, SLC7A5 integrates extracellular BCAAs levels with intracellular metabolic and signaling rewiring across immune cell types, positioning it as a promising therapeutic target for reprogramming antitumor immunity.

## 7 BCAAs and epigenetic regulation in cancer

Epigenetic dysregulation-including DNA methylation, histone modifications, and RNA methylation-plays a key role in cancer (Esteller, 2008; Goldberg et al., 2007). METTL16 regulates *BCAT1/2* expression via m^6^A modification, reprogramming BCAAs metabolism to support tumorigenesis (Zou et al., 2019). In AML, *BCAT1* overexpression or IDH mutation reduces α-KG levels, inhibiting TET enzymes and causing DNA hypermethylation (Hattori et al., 2017; Raffel et al., 2017). In HCC, BCAT1 upregulation correlates with promoter hypomethylation (Zou et al., 2019). In lung cancer and leukemia, *BCAT1* expression is suppressed by H3K9me2/3 (via G9a/SUV39H1) and H3K27me3 (via EZH2) (Wang et al., 2019; Gu et al., 2019). BCAAs metabolism fuels tumorigenesis through epigenetic mechanisms: METTL16 upregulates *BCAT1/2* via m^6^A modification; BCAT1 or IDH mutations deplete α-KG, causing DNA hypermethylation; and repressive histone marks suppress BCAT1 in lung cancer.

BCAAs-derived acetyl-CoA contributes to histone acetylation, influencing gene expression. Leucine-derived acetyl-CoA promotes tumor growth, while lysine-derived acetyl-CoA supports self-renewal in CRC cells (La Vecchia and Sebastián, 2020). Intriguingly, a low-BCAAs diet reduces histone acetylation and delays aging in *Drosophila*, reversible by isoleucine supplementation (Weaver et al., 2023). Given the shared epigenetic mechanisms between aging and cancer, BCAAs-mediated epigenetic regulation represents a promising research avenue. BCAAs-derived acetyl-CoA drives histone acetylation and tumor growth. Leucine and lysine support cancer progression, while a low-BCAAs diet delays aging through reduced histone acetylation, suggesting shared epigenetic pathways in cancer and aging.

## 8 Dietary BCAAs interventions in cancer therapy

The potential of branched-chain amino acid (BCAAs) supplementation as an adjunct to cancer therapy is characterized by conflicting evidence, revealing a context-dependent relationship that varies by tumor type and clinical setting.

In colorectal cancer (CRC), findings appear divergent. While one investigation reported a positive association between higher dietary BCAAs intake and all-cause mortality in CRC patients (Long et al., 2021), this contrasts with data from large-scale case-control and major US cohort studies, which have indicated an inverse association between BCAAs intake and the risk of sigmoid colon cancer specifically (Rossi et al., 2021; Katagiri et al., 2020). This discrepancy highlights the need for subtype-specific analyses.

A more consistent pro-tumorigenic role for BCAAs has been observed in pancreatic cancer models. Both clinical epidemiology and preclinical experimentation align in this context. An Italian multicenter case-control study identified a positive correlation between dietary BCAAs intake and pancreatic cancer risk in humans (Rossi et al., 2023). Correspondingly, in genetically engineered LSL-KrasG12D/+; Pdx1-Cre (KC) mice-a model of pancreatic carcinogenesis--a BCAAs-enriched diet was shown to accelerate the progression of pancreatic intraepithelial neoplasia (PanIN) (Li J. T. et al., 2022).

Conversely, in breast cancer, evidence points toward a potential protective or therapeutic effect of BCAAs. Epidemiological data suggest an inverse correlation between dietary BCAAs intake and the risk of postmenopausal breast cancer (Nouri-Majd et al., 2022). This observation is supported by mechanistic studies demonstrating that elevated BCAAs concentrations *in vitro* impair the migration and invasion capacities of breast cancer cells, an effect potentially mediated by N-cadherin downregulation. Furthermore, *in vivo*, a high-BCAAs diet was found to suppress both primary tumor growth and lung metastasis in mouse models, suggesting a role for BCAAs supplementation in inhibiting breast cancer progression (Chi et al., 2022). However, Assessment of long-term dietary BCAAs intake did not support an association with invasive breast cancer risk, overall or for any individual BCAAs (Tobias et al., 2021).

Beyond direct antitumor effects, BCAAs supplementation demonstrates considerable utility in supportive oncology care, particularly in hepatic malignancies. Perioperative administration of BCAAs has been associated with a reduced incidence of postoperative complications, including ascites and infections, in patients undergoing cancer surgery (Cogo et al., 2021). More specifically, in hepatocellular carcinoma (HCC) patients receiving locoregional therapies (e.g., radiofrequency ablation, transarterial chemoembolization), BCAAs supplementation has been shown to improve nutritional and metabolic parameters-such as serum albumin levels and the non-protein respiratory quotient (npRQ)-while also enhancing quality of life. Importantly, this supportive intervention is linked to improved clinical outcomes, including a reduction in Child-Pugh score, lower recurrence rates, and prolonged overall survival (Sideris et al., 2023). These benefits suggest that BCAAs may effectively mitigate adverse effects associated with conventional anticancer treatments, including surgery, chemotherapy, and radiotherapy.

In summary, the impact of BCAAs supplementation is not uniform across cancers, exhibiting pro-tumor effects in pancreatic models, potential inhibitory effects in breast cancer, and significant supportive benefits in the management of HCC. This tissue- and context-specificity underscores the necessity for precise, indication-driven application in clinical practice.

## 9 Targeted BCAAs inhibitors in cancer

The accumulation and dietary intake of Branched-chain amino acids (BCAAs) have been shown to modulate tumor cell proliferation, overall tumor burden, and patient survival. Consequently, key components of BCAAs handling-including specific transporters and catabolic enzymes such as BCAT1, BCAT2, and BCKDK-are increasingly regarded as potential therapeutic targets in oncology. Several classes of inhibitors directed against these enzymes have subsequently been identified and characterized (Table 4).

**TABLE 4:** Pharmacological targeting of the BCAAs metabolic network: inhibitors of BCAT1, BCAT2, and BCKDK.

Target	Inhibitor class/Compound	Primary mechanism/Key features	References
BCAT1	N-Arylcarbonylarylsulfonylhydrazides	Neuroprotective; Reduce ROS Preserve mitochondrial function	Hu et al. (2006)
BCAT1	Thiazolones	Preventing neuronal loss	Li et al. (2004)
BCAT1	4-methyl-5-oxohexanoic acid (MOHA)	Downregulates CD147 Inhibits MMP2, Suppresses proliferation	Papatthanassiu, (2012)
BCAT1	Gabapentin and analogs	Selective, Competitive BCAT1 inhibitors	Kukkar et al. (2013), Chen et al. (2022), Radlwimmer et al. (2013), Grankvist et al. (2018)
BCAT1	Bufalin	Directly binds BCAT1 Sensitizes cells togemcitabine/5-FU	Zhang et al. (2025b)
BCAT1	WQQ-345	Novel bridged-bicyclic scaffold Active againsthigh-BCAT1 cells	Luo et al. (2025)
BCAT1	MiR-320a	Epigenetic regulator; Low levels correlate with BCAT1 upregulation; Restoration suppresses tumor	Zhao et al. (2024)
BCAT1	Curcumin	Downregulates BCAT1 Inhibits mTOR signaling	Tseng et al. (2021)
BCAT1	Candesartan	Dose-dependently inhibits wild-type and mutant BCAT1 (E61A); Impairs migration & RhoC activity	Qian et al. (2023)
BCAT2	Fused imidazoles with cis-1,3-cycloalkanedi-amine scaffolds	Reduce BCAT2 levels	Deng et al. (2015)
BCAT2	Biphenyl-tetrahydropyrrole (12-15e)	Potent BCAT2 inhibitor identified from DNA-encoded library screening	Deng et al. (2015)
BCAT2	Dihydropyrazolopyrimidine (13–61)	Orally available; raises plasma BCAAs dose-dependently via BCAT2 inhibition	Deng et al. (2015)
BCAT2	Thiophenopyrimidine carboxamides	Micromolar inhibition detectable cellular activity, favorable solubility	Deng et al. (2015)
BCAT2	Telmisartan	Concentration-dependently suppresses BCAT2 activity; may modulate lipid metabolism via PPAR	Zhang et al. (2025b)
BCAT2	Pyrazoline derivatives (16-4a, 16-4e, 16-4f)	Effective binding to hBCAT2 active site (molecular docking)	Ray et al. (2023)
BCKDK	BT2	Allosteric modulator; Induces conformational change, Dissociates BCKDK from complex, Promote degradation	Tso et al. (2014)
BCKDK	(S)-α-chlorophenylpropionate	Allosteric inhibitor; Similar mechanism to BT2	Tso et al. (2014)
BCKDK	PPHN & POAB	Novel allosteric compounds identified via virtual screening Potent inhibition, Anti-proliferative & Pro-apoptotic	Li Z. et al. (2024)
BCKDK	PF-07328948	Optimized BT2 derivative via 3-aryl substitution Degrade BCKDK, Improves metabolic and cardiac parameters	Filipski et al. (2025)
BCKDK	GSK180736A	BCKDK inhibitor; Overcomes resistance when combined with DNA-damaging chemotherapeutics	Liu et al. (2025)
BCKDK	Tetrazole-containing scaffolds	Bind BCKDK at multiple sites (fragment-based screening)	Liu et al. (2023)
BCKDK	Certain angiotensin receptor blocker	Identified as potent BCKDK inhibitors via structure-based virtual screening	Liu et al. (2023)
BCKDK	Bicyclic carboxyamide inhibitors	Bioactive; Expand chemical space for targeting BCKDK	Liang et al. (2024)

### 9.1 BCAT1 inhibitors

BCAT1, which is highly expressed in neuronal tissues and contributes to glutamate synthesis can be inhibited by several compound classes *N-Arylcarbonylarylsulfonylhydrazides* function as BCAT1 inhibitors and have been explored as neuroprotective agents in neurodegenerative contexts. Their mechanism extends to the preservation of mitochondrial function, achieved by mitigating oleic acid–induced ROS generation and membrane potential loss, thereby reducing autophagic flux and suppressing associated airway inflammation and remodeling (Hu et al., 2006). Thiazolones represent another class of BCAT1 inhibitors primarily investigated for preventing neuronal loss, though their potential application in cancer therapy remains to be elucidated (Li et al., 2004). Among compounds evaluated in oncology, 4-methyl-5-oxohexanoic acid (MOHA) has been identified as a BCAT1 inhibitor with antitumor activity. In MDA-MB-231 breast cancer cells, MOHA downregulates surface CD147 expression, attenuates immune reactivity, and inhibits proliferation. It also suppresses the growth of other cancer lines, including MCF-7, MDA-MB-435, and HT-29, while reducing levels of matrix metalloproteinase-2 (MMP2) (Papatthanassiu, 2012). The widely used neuroactive agent gabapentin-along with its analogs-acts as a selective, competitive inhibitor of BCAT1 and has been proposed for the treatment of glioblastoma and astrocytoma (Kukkar et al., 2013; Chen et al., 2022; Radlwimmer et al., 2013). Interestingly, however, studies in HCT116 colorectal cancer cells revealed that gabapentin-mediated growth suppression occurs independently of BCAAs transamination, pointing to an off-target effect possibly involving mitochondrial BCKA catabolism (Grankvist et al., 2018). This suggests that gabapentin may exert context-dependent antitumor effects through both BCAT1-dependent and -independent pathways. Beyond conventional small molecules, several other agents modulate BCAT1 activity in cancer settings. Bufalin, a broad-spectrum anticancer compound known to regulate PI3K/Akt, Wnt/β-catenin, and NF-κB signaling, has recently been shown to bind directly to BCAT1, thereby sensitizing pancreatic cancer cells to gemcitabine and 5-fluorouracil (Zhang W. et al., 2025). The GABA-derived molecule WQQ-345, featuring a unique bridged bicyclic scaffold, exhibits *in vitro* and *in vivo* activity against TKI-resistant lung cancer cells with high *BCAT1* expression (Luo et al., 2025). Epigenetic regulation also plays a role: low circulating levels of miR-320a in patients with growth hormone–secreting pituitary neuroendocrine tumors correlate with BCAT1 upregulation, and restoration of this microRNA suppresses tumor progression via BCAT1 targeting (Zhao et al., 2024). Furthermore, curcumin induces apoptosis in cytarabine-resistant AML cells partly through downregulation of BCAT1 and inhibition of mTOR signaling (Tseng et al., 2021). The AT1R antagonist candesartan dose-dependently inhibits both wild-type and mutant BCAT1 (E61A), impairs migration and Ras Homolog Gene Family, Member C (RhoC) activity in esophageal and gastric cancer cells, and suppresses peritoneal metastasis in a RhoC-dependent manner (Qian et al., 2023).

### 9.2 BCAT2 inhibitors

Inhibition of BCAT2 has been pursued using structurally diverse compounds. Fused imidazoles containing cis-1,3-cycloalkanedi-amine scaffolds-such as benzimidazoles, pyridinoimidazoles, and pyrimidinoimidazoles-effectively reduce BCAT2 levels in engineered A549 cells (Deng et al., 2015). Through screening of DNA-encoded libraries encompassing 14 billion compounds, Deng et al. identified biphenyl-tetrahydropyrrole (12-15e) as a potent BCAT2 inhibitor active after DNA cleavage (Deng et al., 2015). Another orally available agent, dihydropyrazolopyrimidine with benzylamine (13–61), elevates plasma BCAAs concentrations in a dose-dependent manner via BCAT2 inhibition. Additional BCAT2-directed molecules include thiophenopyrimidine carboxamides, which show micromolar inhibition, detectable cellular activity, and favorable solubility. The clinically used angiotensin receptor blocker telmisartan concentration-dependently suppresses BCAT2 enzymatic activity in biochemical and cellular assays and may promote lipid metabolism through BCAT2-mediated PPAR activation, suggesting potential utility in metabolic disorders (Zhang et al., 2025b). Finally, Ray and colleagues designed a series of pyrazoline derivatives, among which compounds 16-4a, 16-4e, and 16-4f demonstrated effective binding to the BCAT2 active site in molecular docking studies (Ray et al., 2023). Together, these findings underscore the pharmacologic tractability of BCAAs catabolic enzymes and highlight a growing repertoire of chemical tools and drug candidates for potentially modulating BCAT1 and BCAT2 in cancer and related pathologies.

### 9.3 BCKDK inhibitors

Branched-chain α-ketoacid dehydrogenase kinase (BCKDK) plays a multifaceted role in tumorigenesis, notably through phosphorylation of MEK to activate the RAS/RAF/MEK/ERK signaling axis, thereby promoting cellular proliferation (Xue et al., 2017). Given its central regulatory function, BCKDK has emerged as a compelling therapeutic target in oncology. Pharmacological inhibition of BCKDK, achieved via small-molecule allosteric modulators such as 3,6-dichloro-1-benzothiophene-2-carboxylic acid (BT2) and (S)-α-chlorophenylpropionate, disrupts its activity by inducing conformational shifts within the N-terminal domain helix (Tso et al., 2014). This structural alteration triggers the dissociation of BCKDK from the BCKDH complex and facilitates its subsequent degradation, ultimately suppressing tumor growth (Du et al., 2022; East et al., 2021). Comparative analyses indicate that BT2 possesses superior inhibitory potency and metabolic stability relative to (S)-α-chlorophenylpropionate, alongside an ability to enhance BCKDH activity in primary hepatocytes (East et al., 2021).

The therapeutic potential of BCKDK inhibition has been validated across diverse malignancies. In triple-negative breast cancer models, intervention targeting BCKDK lowers branched-chain ketoacid levels, remodels BCAAs metabolic flux, and concurrently upregulates Sestrin2 while suppressing mTORC1 signaling-a combination that attenuates proliferation and induces apoptotic cell death. Synergy with doxorubicin has further been documented (Biswas et al., 2021). Similarly, in ovarian cancer and non-small cell lung cancer, *BCKDK* expression correlates with disease progression, and its genetic or pharmacological inhibition curbs cancer cell proliferation, migration, and cell cycle progression (Li H. et al., 2022; Bagheri et al., 2022). Continuous optimization of inhibitory strategies is underway. Leveraging virtual screening and structural biology, novel allosteric compounds including PPHN and POAB have been identified, demonstrating potent BCKDK inhibition coupled with marked anti-proliferative and pro-apoptotic activities both *in vitro* and *in vivo* (Li C. et al., 2024). One particularly optimized derivative, PF-07328948, was developed through strategic modification of the BT2 core via 3-aryl substitution, capitalizing on a cryptic binding pocket. This agent effectively degrades BCKDK *in situ*, markedly reduces circulating BCAAs and BCKA concentrations, exhibits favorable pharmacokinetics, and improves both metabolic parameters and cardiac function endpoints in rodent models of heart failure (Filipski et al., 2025).

Beyond its metabolic roles, BCKDK participates in DNA damage response pathways, including homologous recombination repair. Co-administration of BCKDK inhibitors such as GSK180736A with DNA-damaging chemotherapeutics can therefore overcome treatment resistance in breast cancer models (Liu et al., 2025). In parallel, fragment-based screening employing NMR and computational methods has identified tetrazole-containing scaffolds that bind BCKDK at multiple sites. Subsequent structure-based virtual screening revealed that certain angiotensin receptor blocker antihypertensives and related compounds also act as potent BCKDK inhibitors, suggesting a novel therapeutic avenue that merges BCKDK inhibition with cardiovascular management in conditions like heart failure (Liu et al., 2023). Reports of bioactive bicyclic carboxyamide inhibitors further underscore the expanding chemical space for targeting this kinase (Liang et al., 2024). Collectively, these advances highlight BCKDK and associated pathways-including the USP1/BCAT2 axis-as precision oncology targets with translational promise across several disease contexts.

It should be noted, however, that current inhibitors present certain pharmacological challenges. BT2, for instance, acts as a lipophilic weak acid capable of mitochondrial uncoupling, which may lead to off-target effects. Its high affinity for plasma albumin also potently reduces systemic tryptophan levels, a phenomenon that warrants consideration in therapeutic development.

### 9.4 Therapeutic promise and translational hurdles

Building upon prior mechanistic insights, the translational targeting of branched-chain amino acid (BCAAs) metabolism-especially through inhibition of BCAT1, BCAT2, and BCKDK-reveals considerable therapeutic potential alongside substantial complexity.

Concerning inhibitor development, although multiple chemical scaffolds have been explored-from gabapentin and its analogs against BCAT1 to allosteric modulators such as BT2 for BCKDK-most remain in preclinical or early discovery stages (Kukkar et al., 2013; Tso et al., 2014; Li C. et al., 2024). Exceptions include repurposed clinically approved agents like the AT1R antagonist candesartan and the neuroactive drug gabapentin, which exhibit direct BCAT1 inhibitory activity in cancer models and may allow accelerated clinical evaluation (Qian et al., 2023; Kukkar et al., 2013). Nevertheless, a dedicated, clinical-grade inhibitor designed specifically for oncology applications has not yet been developed, underscoring a persistent disconnect between target validation and drug discovery.

A central translational concern involves potential effects on normal tissues. BCAAS metabolism is integral to physiological homeostasis, particularly in the brain and skeletal muscle. BCAT1, for example, supports glutamate synthesis in neuronal tissue, raising the possibility of neurotoxicity with systemic inhibition (Dimou et al., 2022). Likewise, BCAT2 plays a key role in nitrogen and energy balance in muscle. Consequently, establishing a therapeutic window will likely require tumor-selective delivery approaches-such as nanoparticle conjugates or protease-activated prodrugs-or the identification of isoform- or context-selective inhibitors that preserve essential physiological functions.

Several key challenges hinder clinical translation. First, metabolic plasticity within tumors and a dynamic tumor microenvironment may enable compensatory pathways or alternative nutrient acquisition, leading to inherent or adaptive resistance. Second, the absence of validated predictive biomarkers represents a major obstacle to identifying patients whose tumors display genuine dependence on BCAAs pathways. Putative biomarkers, including BCAT1 promoter methylation, circulating levels of branched-chain ketoacids (BCKAs), or specific transcriptional signatures, require rigorous clinical validation (Wegermann et al., 2018; Zou et al., 2019). Third, achieving favorable pharmacokinetics and target selectivity with current lead compounds, while limiting on-target toxicity in normal tissues, remains a demanding pharmacological challenge.

Pharmacological modulation may also provoke unintended off-target or compensatory responses. For instance, gabapentin’s anti-proliferative effects in certain settings appear independent of BCAT1 inhibition and may involve off-target mitochondrial actions (Grankvist et al., 2018). The BCKDK inhibitor BT2, though effective, functions as a lipophilic weak acid capable of mitochondrial uncoupling-a property that could influence both its efficacy and adverse effect profile (East et al., 2021). Moreover, systemic BCAT2 inhibition would be expected to increase circulating BCAAs, which might inadvertently support tumor growth in contexts where BCAAs uptake is not limiting, or induce unanticipated systemic metabolic alterations.

In summary, while BCAAs-catabolizing enzymes remain attractive therapeutic targets, their successful incorporation into oncology demands a multifaceted approach. This includes the rational design of next-generation inhibitors with improved selectivity, the parallel development of reliable companion diagnostics, and the proactive addressing of metabolic adaptation through rationally designed combination regimens. Navigating these translational subtleties will be critical to transforming the compelling preclinical data on BCAAs metabolism into precise and clinically viable cancer therapies.

## 10 Conclusion and future perspectives

Branched-chain amino acid (BCAAs) metabolism, once viewed primarily through the lens of energy production and protein synthesis, is now recognized as a central signaling node and a critical driver of metabolic reprogramming in cancer. Its role in tumor progression is multifaceted, encompassing not only the provision of biosynthetic precursors but also the regulation of key oncogenic pathways-including mTOR and PI3K/AKT-the modulation of epigenetic programs, and the active contribution to an immunosuppressive tumor microenvironment (TME). Enzymes such as BCAT1, BCAT2, and BCKDK, which govern this metabolic network, thus represent promising albeit complex therapeutic targets. Translating this mechanistic understanding into clinical advancement necessitates a coordinated strategy addressing several interdependent fronts.

A primary challenge lies in the development of inhibitors with high isoform selectivity and tumor-specific delivery. Research efforts must prioritize the creation of clinical-grade compounds targeting BCAT1 and BCKDK, coupled with refined delivery mechanisms designed to minimize on-target toxicity in healthy tissues-a hurdle that has historically limited the therapeutic window of metabolism-targeting agents.

Closely linked to this therapeutic aim is the urgent need to identify predictive biomarkers. The deployment of comprehensive multi-omics profiling across diverse malignancies will be essential to define reliable indicators-for instance, BCAT1 promoter methylation status, circulating branched-chain ketoacid (BCKA) concentrations, or specific transcriptional subtypes-that can stratify patient populations whose tumors are truly dependent on BCAAs metabolism and are therefore more likely to respond to its inhibition.

The physiological relevance of preclinical models also requires critical reassessment. Traditional cell lines and xenograft systems often fail to recapitulate the metabolic interplay and immune context of human tumors. A shift toward more integrated models, such as patient-derived organoids, immunocompetent genetically engineered mouse models (GEMMs), and humanized mouse models, would provide a more accurate platform for evaluating drug efficacy and understanding TME-specific metabolic crosstalk.

Moreover, the systemic consequences of prolonged BCAAs pathway inhibition remain poorly characterized. Potential impacts on whole-organism physiology-including muscle homeostasis, systemic glucose regulation, and immune function-must be rigorously investigated to anticipate and manage possible side effects in future clinical applications.

Ultimately, the clinical evaluation of these agents will depend on innovative trial design. Well-controlled, early-phase studies that incorporate biomarker-driven patient stratification are imperative to assess the safety and preliminary efficacy of BCAAs pathway inhibitors, both as monotherapies and in rational combination with existing modalities like chemotherapy, radiotherapy, or immunotherapy.

By deciphering the context-dependent roles of BCAAs metabolism in cancer, fundamental insights into tumor biology will be gained, while simultaneously paving the way for novel, metabolism-directed therapeutic strategies with the potential to improve patient outcomes.
